# An analysis of intestinal morphology and incretin-producing cells using tissue optical clearing and 3-D imaging

**DOI:** 10.1038/s41598-022-22511-7

**Published:** 2022-10-20

**Authors:** Tomonobu Hatoko, Norio Harada, Shinsuke Tokumoto, Shunsuke Yamane, Eri Ikeguchi-Ogura, Tomoko Kato, Takuma Yasuda, Hisato Tatsuoka, Satoko Shimazu-Kuwahara, Daisuke Yabe, Yoshitaka Hayashi, Nobuya Inagaki

**Affiliations:** 1 Department of Diabetes, Endocrinology and Nutrition, Kyoto University Graduate School of Medicine, 54 Kawahara-cho, Shogoin, Sakyo-ku, Kyoto, 606-8507 Japan; 2 Preemptive Medicine and Lifestyle Related Disease Research Center, Kyoto University Hospital, Kyoto, Japan; 3 Department of Diabetes and Endocrinology, Graduate School of Medicine, Gifu University, Gifu, Japan; 4 Division of Stress Adaptation and Protection, Department of Endocrinology, Research Institute of Environmental Medicine, Nagoya University, Nagoya, Japan

**Keywords:** Anatomy, Endocrinology, Gastroenterology

## Abstract

Tissue optical clearing permits detailed evaluation of organ three-dimensional (3-D) structure as well as that of individual cells by tissue staining and autofluorescence. In this study, we evaluated intestinal morphology, intestinal epithelial cells (IECs), and enteroendocrine cells, such as incretin-producing cells, in reporter mice by intestinal 3-D imaging. 3-D intestinal imaging of reporter mice using optical tissue clearing enabled us to evaluate both detailed intestinal morphologies and cell numbers, villus length and crypt depth in the same samples. In disease mouse model of lipopolysaccharide (LPS)-injected mice, the results of 3-D imaging using tissue optical clearing in this study was consistent with those of 2-D imaging in previous reports and could added the new data of intestinal morphology. In analysis of incretin-producing cells of reporter mice, we could elucidate the number, the percentage, and the localization of incretin-producing cells in intestine and the difference of those between L cells and K cells. Thus, we established a novel method of intestinal analysis using tissue optical clearing and 3-D imaging. 3-D evaluation of intestine enabled us to clarify not only detailed intestinal morphology but also the precise number and localization of IECs and incretin-producing cells in the same samples.

## Introduction

The intestine is an important organ involved in digestion, absorption, and energy metabolic regulation such as appetite regulation and nutrient accumulation through various intestinal hormones^1^. Intestinal epithelial cells (IECs) are located at the boundary between the intestine and intestinal lumen and are composed of several types of cells such as absorptive cells, goblet cells, Paneth cells, and enteroendocrine cells (ECs)^2^. Two major incretins, glucagon-like peptide-1 (GLP-1) and glucose-dependent insulinotropic polypeptide/gastric inhibitory polypeptide (GIP), are secreted from enteroendocrine L cells and K cells, respectively, in response to nutrient ingestion^3–5^, and have various biological effects such as enhancing glucose-dependent insulin secretion from pancreatic β-cells^6–8^ and regulating body weight^9–13^. Thus, GLP-1 and GIP play a crucial role in glucose and body weight control.

Immunohistological analysis of intestinal tissue sections generally has been used for evaluating the number and localization of target cells in intestine^14^. However, it is difficult to envision the three-dimensional (3-D) structure of villi and crypts and to evaluate the number and localization of IECs and ECs under two-dimensional (2-D) intestinal tissues.

Tissue optical clearing is an observational method that has shown remarkable progress in recent years. Clear, unobstructed brain/body imaging cocktails and computational analysis (CUBIC) protocol were established in 2014, making the whole body transparent in mice and enabling detailed evaluation of each organ’s 3-D structure and its cells by tissue staining and autofluorescence^15–17^. Thus, CUBIC protocol may facilitate not only visualization of 3-D intestinal imaging but also 3-D intestinal structure evaluation^18,19^.

In the present study, we succeeded in obtaining detailed 3-D intestinal imaging of reporter mice using the CUBIC protocol and analyzing intestinal morphology, IECs, and ECs such as incretin-producing cells.

## Materials and methods

### Animals

Villin, encoded by *Villin1*, is an actin binding protein expressed throughout the crypt-villus axis in small intestine and colon of mice^20,21^. *Villin1-Cre* transgenic mice and *Ai14* mice were previously generated (JAX stock #004586, #007908) (Jackson Laboratory, Bar Harbor, ME)^21,22^. *Villin1-Cre* and *Ai14* heterozygous (Villin1-Tomato) mice, which enable visualization of IECs by tdTomato fluorescence, were generated by crossbreeding *Villin1-Cre* transgenic mice and *Ai14* homozygous mice. Analysis of intestinal morphology and IECs was performed using 13-week-old male Villin1-Tomato mice.

*Glucagon*-*green fluorescent protein* (*GFP*) knock-in (Gcg-GFP) heterozygous mice and *GIP*-*GFP* knock-in (GIP-GFP) heterozygous mice, which enabled visualization of L cells and K cells, respectively, were previously generated^23,24^. Villin1-Tomato and Gcg-GFP or GIP-GFP heterozygous (Villin1-Tomato+ Gcg-GFP or Villin1-Tomato+ GIP-GFP) mice, which enabled visualization of both IECs by tdTomato fluorescence and L cells and K cells by GFP fluorescence, were generated by crossbreeding Villin1-Tomato mice and Gcg-GFP or GIP-GFP homozygous mice. Analysis of incretin-producing cells was performed using 13-week-old male Villin1-Tomato+ Gcg-GFP or Villin1-Tomato+ GIP-GFP mice. All mice had free access to standard rodent chow and were housed in a temperature-controlled room with a light–dark cycle of 14:10 h. All animal experiments were performed in compliance with ethical regulations in Kyoto University. Animal care and procedures were approved by the Animal Care and Use Committee of Kyoto University Graduate School of Medicine (Medkyo18245). All animals were handled in accordance with the ARRIVE guidelines.

### Collection of intestine samples and tissue clearing

Mice were anesthetized with intraperitoneal injection of pentobarbital sodium (10 ml/kg) and transcardially perfused with ice-cold phosphate-buffered saline (PBS) followed by ice-cold 4% (w/v) paraformaldehyde (PFA) (Wako Pure Chemical Industries, Osaka, Japan) after insertion of a 23-gauge needle into the left ventricle. The small intestine and the colon of all mice were excised. A total of five samples of about 1 cm of small intestine were collected, one each from the oral side (S1) and the anal side (S5), one from the intermediate area between S1 and S5 (S3) and one each from the intermediate areas between S1 and S3 (S2) and between S3 and S5 (S4) of the small intestine (Supplemental Fig. 1A). S1, S2–S3, and S4–S5 corresponded to duodenum, jejunum and ileum, respectively. A total of three samples of colon were collected, one from the oral side (C1), center (C2) and anal side (C3) of the colon. All samples were immediately immersed in PFA at 4 °C with gentle shaking overnight and washed three times for more than 2 h each in PBS at room temperature with gentle shaking. All samples were immersed in 50% (v/v) CUBIC-L (Tokyo Chemical Industry Co., Ltd, Tokyo, Japan) reagent (1: 1 mixture of water: CUBIC-L) at 37 °C with gentle shaking for 24 h followed by CUBIC-L at 37 °C with gentle shaking for 24 h. After washing in PBS, the samples were immersed in 5 ng/ml 4′,6-diamidino-2-phenylindole (DAPI) (Dojindo Laboratories, Kumamoto, Japan) in PBS at room temperature with gentle shaking for 30 min followed by washing in PBS. The samples were immersed in 50% (v/v) CUBIC-R+ (Dojindo Laboratories) reagent (1: 1 mixture of water: CUBIC-R+) for 24 h followed by immersion in CUBIC-R+ at room temperature with gentle shaking for 24 h.

### Lipopolysaccharide (LPS)-induced intestinal injury and infection model mice

13-week-old male Villin1-Tomato mice were injected intraperitoneally with 10 mg/kg LPS from *E. coli* O111:B4 (Sigma-Aldrich, St. Louis, MO) in saline or an equivalent volume of saline alone^25–27^. After 24 h of injection, the mice were anesthetized and transcardially perfused with the methods described above. Five samples of small intestine and three samples of colon in all mice were collected. In the 3-D imaging group, all samples in each five LPS-injected mice and saline-injected mice (control mice) were cleared by CUBIC methods. In the 2-D imaging group, all samples in each three LPS-injected mice and control mice were fixed in 4% (w/v) PFA solution, embedded in paraffin, and stained with hematoxylin and eosin (HE).

### Image acquisition

3-D fluorescence images were acquired by spinning disk confocal microscopy (Dragonfly, Andor Technology Ltd., Belfast, UK) on an IX83 (Olympus Corp. Tokyo, Japan) device through a UCPLFLN 20× objective lens (Olympus, numerical aperture [NA], 0.7) using a 405 nm laser for DAPI staining (blue), a 488 nm laser for GFP fluorescence imaging (green), and a 561 nm laser for tdTomato fluorescence imaging (red). Data were collected in Spinning Disk 40 μm pinhole mode on a scientific complementary metal oxide semiconductor (sCMOS) camera (Zyla4.2Plus USB3) (Andor Technologies), which had a measured pixel size of 0.95 μm $\times$ 0.95 μm. Using the Z scan mode, each sample was scanned every 2.5 μm in small intestine and colon. Acquired microscopic images were further processed by the deconvolution algorithm for 3D volume reconstruction. 2-D images were acquired by fluorescent microscopy with the FSX100 system (Olympus Corp., Tokyo, Japan).

### Imaging processing

Acquired 3-D fluorescent images were analyzed using Imaris Version 9.1.2 (Bitplane AG, Zurich, Switzerland). From each image, ten villi and crypts were randomly selected in the small intestine and colon, respectively, excepting “winding villi”, which were not completely visible in the 3-D image. “Normalize layers” in Imaris, which is an image processing function for adjusting brightness and contrast of individual slices to a uniform level, was applied to all images. “Surface system” in Imaris was first used to extract only tdTomato-positive cells in villi and crypts, which eliminates cells in villi and crypts not needed for the analysis, and then to create 3-D-tdTomato fluorescent images by smoothly connecting the extracted cells of all images. As a result, IECs shown in blue and incretin-producing cells shown in green were clearly defined in 3 dimensions. The number of IECs was calculated by counting DAPI fluorescence number in the tdTomato fluorescence area. The number of incretin-producing cells was calculated by counting the GFP fluorescence number in the tdTomato fluorescence area. We automatically obtained those numbers by using Spot algorithm in Imaris. Villus length (μm) and crypt depth (μm) of small intestine were measured from the villus-crypt border to the tip of the villus and to the bottom of the crypt, respectively (Supplemental Fig. 1B). Crypt depth (μm) of colon was measured from the top of the crypt to the bottom of the crypt (Supplemental Fig. 1C). The L cell and K cell localizations in the villi were determined by dividing the distance from the villus-crypt border to the L cell and K cell by villus length (Value of 1.00 and 0.00 indicates tip of villus and villus-crypt border, respectively) (Supplemental Fig. 1B). L cell and K cell localization in the crypts were determined by dividing the distance from the villus-crypt border of small intestine or top of the crypt of colon to the L cell and K cell by crypt depth (Value of 0.00 and − 1.00 indicates villus-crypt border [small intestine] or top of the crypt [colon] and the bottom of the crypt, respectively) (Supplemental Fig. 1B,C). The villi of the small intestine and the crypts of the small intestine and colon were divided into three equal parts, the upper, middle, and lower part. Villi and crypts were cut so that their cross sections were vertical to their extension direction. The width of the major and minor axis (μm) of the cross section at mid-line of the villus length and the crypt depth was measured using “Measurement system” in Imaris. In 2-D images, ten villi and crypts were randomly selected in the small intestine and colon. Villus length (μm) and crypt depth (μm) were measured using images of HE staining.

### Statistical analysis

The results are given as mean ± standard error of the mean (SEM). Several data points of some results that exceeded the mean ± 2 standard deviation (SD) were excluded. Statistical significance and single regression analysis were determined using ANOVA with Tukey test in IBM SPSS version 26 (IBM Corp., Armonk, NY) and *P* values < 0.05 were considered statistically significant. A scatter plot matrix was made using JMP Pro statistical software (version 16.1) (SAS Institute, Cary, NC).

## Results

### Morphology of the small intestine in Villin1-Tomato mice

**Figure 1:**
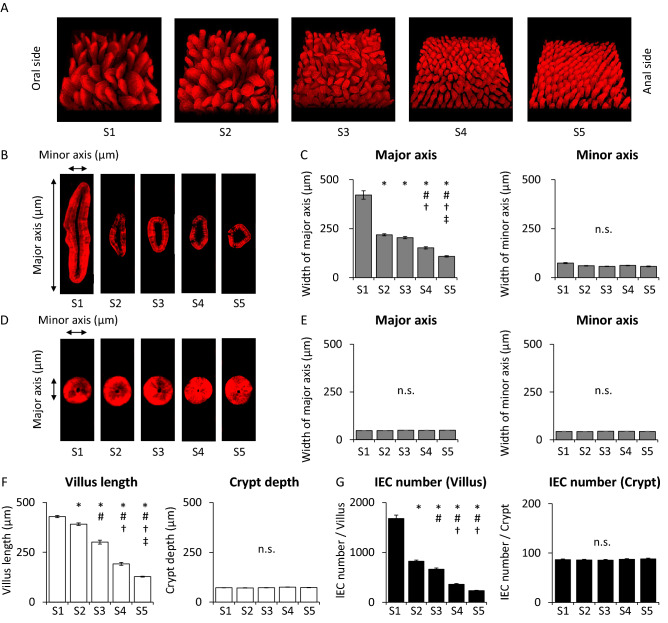
Morphology and analysis of the small intestine in Villin1-Tomato mice. (**A**) The whole images of small intestine and the cross section of (**B**) villi and (**D**) crypts. Width of (**C**) villus and (**E**) crypt major axis and minor axis, (**F**) villus length and crypt depth, and (**G**) intestinal epithelial cell (IEC) number per villus or crypt of each section in small intestine (n = 50 villi or 50 crypts in 5 mice). *P < 0.05 vs. S1, ^#^P < 0.05 vs. S2, ^†^P < 0.05 vs. S3, ^‡^P < 0.05 vs. S4. *n.s.* not significant.

The whole image of each section showed that the size of the villus was greater in the upper small intestine compared with that in the lower small intestine (Fig. 1A). In addition, curved villi were observed mainly in the upper small intestine, whereas upright villi were observed mainly in the lower small intestine. The cross section of villi was spindle-shaped in the upper small intestine and became gradually closer to round shape toward the lower small intestine (Fig. 1B). We then measured the width of the major and minor axis of the villi. Width of villus major axis in S1 was the greatest and that in S2 was halved (Fig. 1C). The width was gradually less from S2 toward S5. The width in S5 was the least of all sections of small intestine. On the other hand, the width of the villus minor axis did not differ in any section of the small intestine. Cross section of all crypts was close to round shape (Fig. 1D) and there was no significant difference in width of major axis and minor axis in all sections of the small intestine (Fig. 1E). Villus length in S1 was the greatest of all of the sections (Fig. 1F). The length gradually shortened from S1 toward S5. IEC number per villus in S1 was the greatest and that in S2 was halved (Fig. 1G). The number was gradually decreased from S2 to S5. The number in S5 was the smallest of all sections of the small intestine. On the other hand, crypt depth and IEC number per crypt did not differ among the sections (Fig. 1F,G).

**Figure 2:**
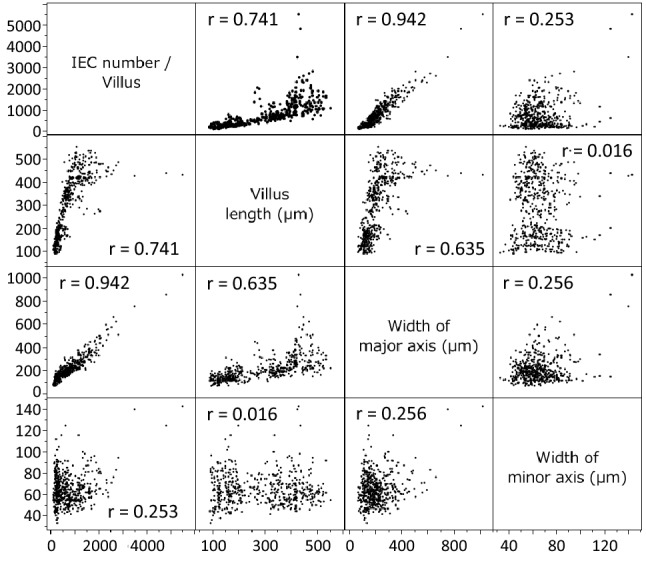
The factors responsible for IEC number in small intestine. A scatter plot matrix showing the relationships among IEC number per villus, villus length, width of major axis, and width of minor axis (n = 50 villi in 5 mice).

We then made a scatter plot matrix to elucidate the stronger factors responsible for IEC number in a villus**,** and found that villus length (r = 0.741, P < 0.01), width of villus major axis (r = 0.942, P < 0.01), and width of villus minor axis (r = 0.253, P < 0.01) were significantly correlated with IEC number per villus (Fig. 2). From these results, the width of the villus major axis was a strong factor for IEC number in a villus, while villus length and width of villus minor axis were moderate and weak factors for IEC number in a villus, respectively.

### Morphology of the colon in Villin1-Tomato mice

**Figure 3:**
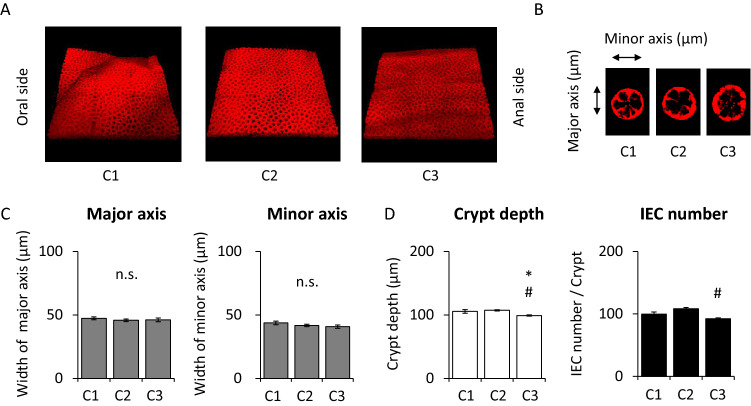
Morphology and analysis of the colon in Villin1-Tomato mice. (**A**) The whole images of colon and (**B**) the cross section of crypts. (**C**) Width of crypt major axis and minor axis, (**D**) crypt depth, and intestinal epithelial cell (IEC) number per crypt of each section in colon (n = 50 crypts in 5 mice). *P < 0.05 vs. C1, ^#^P < 0.05 vs. C2. *n.s.* not significant.

Whole image of each section of colon was similar (Fig. 3A). The cross section of crypts was close to a round shape in all sections of the colon (Fig. 3B). The width of the crypt major and minor axis was not significantly different in the three sections (Fig. 3C). Crypt depth and IEC number per crypt were not significantly different between C1 and C2. Crypt depth in C3 was the shortest in colon and IEC number per villus in C3 was smaller than that in C2 (Fig. 3D).

### Morphology of the small intestine and the colon in LPS-injected mice and control mice

**Figure 4:**
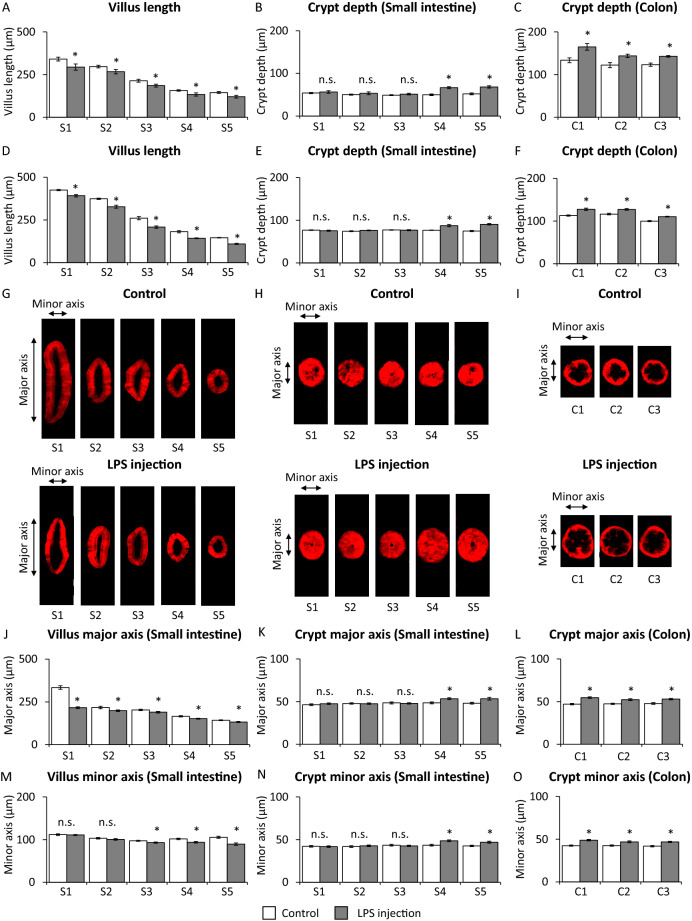
Morphology and analysis of the small intestine and colon in LPS-injected Villin1-Tomato mice. (**A**) Villus length, (**B**) crypt depth of small intestine, and (**C**) crypt depth of colon analyzed by 2-D imaging in control mice (White bar) and LPS-injected mice (Gray bar) (n = 30 villi or 30 crypts in 3 mice). (**D**) Villus length, (**E**) crypt depth of small intestine, and (**F**) crypt depth of colon analyzed by 3-D imaging in control mice (White bar) and LPS-injected mice (Gray bar) (n = 50 villi or 50 crypts in 5 mice). Cross sectional images of (**G**) villus and (**H**) crypt in small intestine and (**I**) crypt in colon of control mice and LPS-injected mice. Width of villus (**J**) major and (**M**) minor axis, of crypt (**K**) major and (**N**) minor axis of small intestine and of crypt (**L**) major and (**O**) minor axis of colon evaluated by 3-D imaging in control mice (White bar) and LPS-injected mice (n = 50 villi or 50 crypts in 5 mice). *P < 0.05 vs. control mice, *n.s.* not significant.

We evaluated morphology of the intestinal disease mouse model in which intestine was injured by LPS injection. In 2-D images, villus length in S1 (duodenum), S2–S3 (jejunum), and S4–S5 (ileum) was shorter in LPS-injected mice than that in control mice (Fig. 4A). Crypt depth in S4–S5 (ileum) was greater in LPS-injected mice than that in control mice (Fig. 4B). Crypt depth in colon was greater in LPS-injected mice than that in control mice (Fig. 4C).

In 3-D images, villus length in S1 (duodenum), S2–S3 (jejunum), and S4–S5 (ileum) was shorter in LPS-injected mice than that in control mice (Fig. 4D). Crypt depth in S4–S5 (ileum) was greater in LPS-injected mice than that in control mice (Fig. 4E). Crypt depth in colon was greater in LPS-injected mice than that in control mice (Fig. 4F). These results show that the data obtained from 3-D images reproduced the data obtained from 2-D images. We then evaluated the width of villus and crypt from 3-D images (Fig. 4G–I). Width of villus major axis in S1 (duodenum), S2–S3 (jejunum), and S4–S5 (ileum) was shorter in LPS-injected mice than that in control mice (Fig. 4G,J), while width of villus minor axis in S3 (part of jejunum) and S4–S5 (ileum) was shorter in LPS-injected mice than that in control mice (Fig. 4M). Width of crypt major and minor axis in S4–S5 (ileum) was longer in LPS-injected mice than that in control mice (Fig. 4H,K,N). Width of crypt major and minor axis in colon was longer in LPS-injected mice than that in control mice (Fig. 4I,L,O).

### L cell number and localization of L cells in villus or crypt of Villin1-Tomato+ Gcg-GFP mice

**Figure 5:**
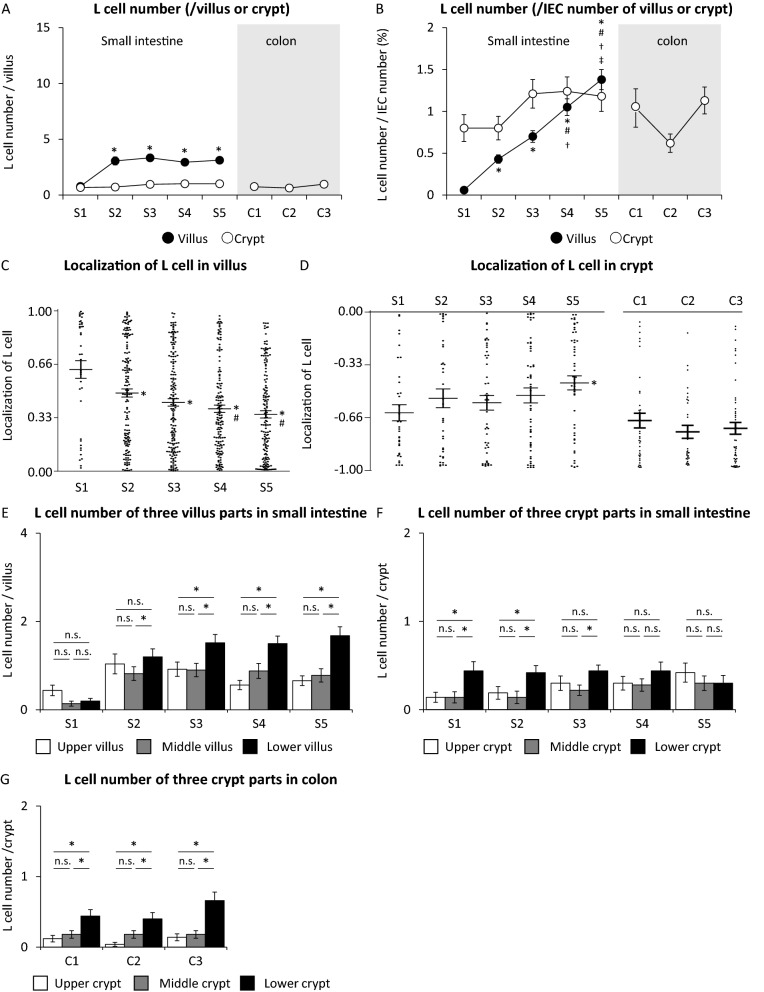
L cell number and localization in villi or crypts of Villin1-Tomato+ Gcg-GFP mice. (**A**) L cell number per villus or crypt and (**B**) L cell number per intestinal epithelial cell (IEC) number in each section of villus and crypt (n = 50 villi or crypts in 5 mice). Black circle and white circle show the number and the percentage of L cells in villus and crypt, respectively. All plots and the average value of L cell localization in (**C**) 50 villi and (**D**) 50 crypts of each section. (**E**) L cell number in the upper, middle, and lower villus of small intestine. (**F**) L cell number in the upper, middle, and lower crypt of small intestine. (**G**) L cell number in the upper, middle, and lower crypt of colon. (n = 50 villi or crypts in 5 mice). *P < 0.05 vs. S1, ^#^P < 0.05 vs. S2, ^†^P < 0.05 vs. S3, ^‡^P < 0.05 vs. S4. *n.s.* not significant.

In villus of small intestine, L cell number per villus in S1 was the least of all sections, and the number was significantly increased in S2 (Fig. 5A). However, there was no significant difference among the four sections in this aspect (S2, S3, S4, and S5). The ratio of L cell number to IEC number in S1 was the smallest of all sections (Fig. 5B). The ratio was gradually increased from S1 toward S5. The ratio in S5 was the largest of all sections. In crypt of small intestine and colon, L cell number per crypt and L cell number per IEC number were not significantly different among the sections (Fig. 5A,B).

Localization of L cells in villus of small intestine was evaluated. The average value of L cell localization in S1 was the highest. The value was gradually reduced from S1 to S5 (Fig. 5C). L cell number was significantly larger in the lower part of the villus than that in the upper and middle parts of the villus in S2–S3 (jejunum) and S4–S5 (ileum) (Fig. 5E). The average value of L cell localization in a crypt was less than 0.5 in S1 to S4 (Fig. 5D). L cell number was significantly larger in the lower part of the crypt than that in the upper and middle parts of the crypt in S1 (duodenum) and S2-S3 (jejunum) (Fig. 5F).

In colon, L cell number per crypt and the ratio of L cell number to IEC number were not significantly different among C1, C2, and C3 (Fig. 5A,B). The average value of L cell localization was around − 0.66 in all three sections (Fig. 5D). L cell number was significantly larger in the lower part of the crypt than that in the upper and middle parts of the crypt in C1, C2, and C3 (Fig. 5G).

### K cell number and localization of K cells in villus or crypt of Villin1-Tomato+ GIP-GFP mice

**Figure 6:**
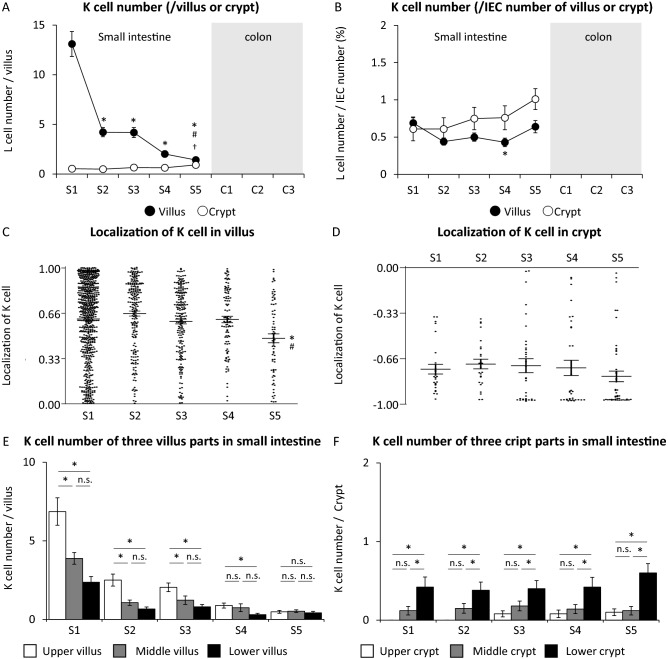
K cell number and localization in villi or crypts of Villin1-Tomato+ GIP-GFP mice. (**A**) K cell number per villus or crypt and (**B**) K cell number per intestinal epithelial cell (IEC) number in each section of villus and crypt (n = 50 villi and crypts in 5 mice). Black circle and white circle show the number and the percentage of K cells in villus and crypt, respectively. All plots and the average value of K cell localization in (**C**) 50 villi and (**D**) 50 crypts of each section. (**E**) K cell number in the upper, middle, and lower villus of small intestine. (**F**) K cell number in the upper, middle, and lower crypt of small intestine. (n = 50 villi or crypts in 5 mice). *P < 0.05 vs. S1, ^#^P < 0.05 vs. S2, ^†^P < 0.05 vs. S3. *n.s.* not significant.

K cells were expressed in small intestine but not in colon (Fig. 6A,B). In villus of small intestine, K cell number per villus in S1 was the largest and that in S2 was dramatically reduced (Fig. 6A). The number was gradually reduced from S3 to S5. The ratio of K cell number to IEC number was not significantly different among the five sections (Fig. 6B). In crypt of small intestine, K cell number per crypt and the ratio of K cell number to IEC number did not differ among the sections (Fig. 6A,B). The average value of K cell localization in villus was around 0.65 in S1, S2, S3, and S4, while the value was 0.50 in S5 (Fig. 6C). The average value of K cell localization in crypt was less than − 0.66 in all sections (Fig. 6D). K cell number was significantly larger in the upper part of the villus than that in the middle and lower parts of the villus in S1 (duodenum) and S2–S3 (jejunum) (Fig. 6E), while K cell number was significantly larger in the lower part of the crypt than that in the upper and middle parts of the crypt in all sections of small intestine (Fig. 6F).

### Association between IEC number and incretin-producing cell number in villi

**Figure 7:**
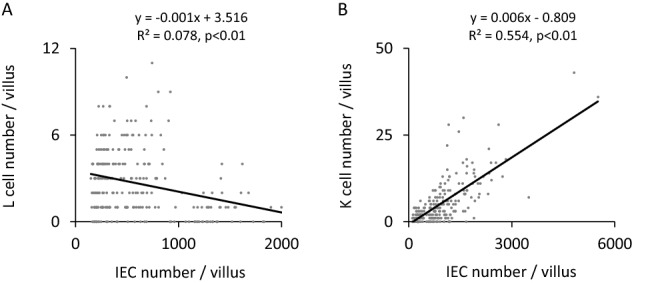
Association between intestinal epithelial cell (IEC) number and incretin-producing cell number in villus. A single regression analysis between IEC number per villus and (**A**) L cell number per villus and (**B**) K cell number per villus (n = 50 villi in 5 mice).

From the results of both GLP-1 and GIP reporter mice, L cell and K cell number were quite different in a villus of small intestine, while the numbers were similar in a crypt of small intestine. We therefore performed a single regression analysis of IEC number and incretin-producing cell number in a villus. The analysis showed that L cell number per villus did not have a specific correlation with IEC number per villus (R^2^ = 0.078, P < 0.01) (Fig. 7A), while K cell number per villus had such a correlation (R^2^ = 0.554, P < 0.01) (Fig. 7B).

## Discussion

Villi are shown by dissecting microscopy to have different morphologies in small intestine^28^. In this study, more detailed morphologies of villi including the villus cross-section have been made possible by tissue optical clearing. IEC number per villus, villus length and crypt depth have previously been evaluated only in longitudinally sectioned images of villi using histological analysis, which could be inaccurate due to the two-dimensionality of the image^29–31^. In this study, we were able to more precisely evaluate parameters by 3-D intestinal imaging. 3-D intestinal imaging using optical tissue clearing enabled us to evaluate both detailed intestinal morphologies and cell numbers, villus length and crypt depth in the same samples. By referring to various results obtained from the same samples, we show that IEC number per villus has a stronger correlation with size of the villus major axis than villus length or size of the villus minor axis. In addition, the 3-D intestinal imaging using the incretin reporter mouse enabled us to evaluate the number and location of incretin-producing cells in the villi and crypts more precisely than immunohistochemistry of intestinal tissue sections. Analysis of 3-D intestinal imaging clearly showed that there is considerable difference in the number and localization of L cells and K cells.

We generated intestinal disease mice by LPS injection in addition to using a non-disease mouse model to validate the 3-D intestinal imaging method with tissue optical clearing. Some reports showed that, in the LPS injection rodent model, villus length is shorter and crypt depth of ileum is greater than that in control model using 2-D images, while there was no data on crypt depth in colon^25–27,32,33^. In this study, the results by 3-D imaging were similar to those found in 2-D images, and were consistent with the data previously reported. In addition, we were able to show the width of cross section of villi and crypts in LPS-injected mice and control mice using 3-D imaging.

Our previous study using GIP reporter mice evaluated K cell number per IEC number in a villus of small intestine by flow cytometry analysis^34^. In that study, K cell number was shown to be larger in the upper small intestine than that in the lower small intestine. On the other hand, using tissue optical clearing in this study, K cell number per IEC number in a villus of small intestine was found to be similar in the upper and the lower small intestine. Moreover, K cell number per IEC number in small intestine was found to be higher in this study (around 0.5% in upper and lower small intestine) than that in the previous report (0.05% and 0.028% in upper and lower small intestine, respectively). There are several possible reasons for these differences. First, GFP-positive cells were shown to have a wide-range of fluorescence intensity, from strong to weak, by tissue optical clearing. GFP-positive cells with weak fluorescence intensity could be captured using tissue optical clearing; those cells might not be captured using flow cytometry. As mentioned above, GFP-positive cell number in the previous report could be less than the actual number of GFP-positive cell. Second, there are many immune cells such as IgA-producing plasma cells and intestinal intraepithelial T lymphocytes inside the villi^35^. In the present study, we were able to count only IECs by counting Tomato-positive cells using tissue optical clearing and IMARIS software; flow cytometry might count not only IECs but also other cells such as immune cells. These two reasons raise the possibility that GFP-positive cell number was found in the previous 2-D report to be smaller and IEC number larger than the actual number.

All IECs including ECs have been shown to move upward for about 60–72 h at a constant rate along the villus from the bottom of the crypt, and to be ejected when they reach the villus tip^36,37^. This process occurs continually in small intestine and promotes IEC replacement and turnover in a “conveyor belt” fashion. In the present study using the 3-D intestinal imaging method, K cells were shown to be expressed more at the upper part of a villus in duodenum, jejunum and part of ileum and at the lower part of a crypt, while L cells were expressed more at the lower part of a villus in jejunum and ileum and at the lower part of a crypt in duodenum, jejunum, and colon. These results show the polarity of both L cells and K cells along the crypt-villus axis. Villus-crypt heterogeneity of enteroendocrine products was characterized in the early 1990s^38^. It was reported that L cells switched from proglucagon-producing cells to secretin-producing cells along the crypt-villus axis and that L cell number was greatly diminished when bone morphogenic protein (BMP) signaling, which increases toward the villus and is highest at the villus tips, was elevated^38–40^. In addition, in a study using organoid derived from small intestine of neurogenin 3 (Ngn3) reporter mice, ECs were expressed only on the crypt side until 80 h after Ngn3 expression, and were observed on both crypt side and villus side after 80 h of Ngn3 expression^41^. Thus, it was suggested that ECs including incretin-producing cells do not move along the epithelial “conveyor belt”. Hormone switching through BMP signaling and the non-constant movement of L cells along the crypt-villus axis could generate the polarity of L cells along the crypt-villus axis, which we showed in this study.

There are several limitations in this study. First, only eight samples were collected from small intestine and colon. While the results of this analysis might be applicable to only a part of the intestine, they may be useful for understanding the tendency toward change of the various parameters in the small intestine and colon. Second, several studies have reported cells producing both GLP-1 and GIP (LK cells)^42,43^. LK cells were not evaluated in this study; L cells and K cells were evaluated separately by GFP fluorescence driven by native mouse glucagon and GIP promoter. Further studies are required to elucidate the characteristics of LK cells. Third, there are presently no reliable data on the fidelity of GLP-1 and GIP expression in Gcg-GFP and GIP-GFP mice, respectively. Our previous reports found by immunostaining that most GFP positive cells were merged with GLP-1 or GIP positive cells^23,24^. Thus, these mice may be useful in this study.

In conclusion, we established a method of intestinal analysis using tissue optical clearing and 3D-imaging. Using this method, we were able to elucidate not only intestinal morphology and IEC number but also the number and localization of target cells in intestine. This method may also be applied to detailed evaluation of intestinal changes induced by various diets and drugs or under pathological conditions.

## Supplementary Information

Supplementary Figure 1.

## Abbreviations

IEC: Intestinal epithelial cell
EC: Enteroendocrine cell
GLP-1: Glucagon-like peptide-1
GIP: Glucose-dependent insulinotropic polypeptide/gastric inhibitory polypeptide
CUBIC: Clear, unobstructed brain/body imaging cocktails and computational analysis
3-D: Three-dimensional
GFP: Green fluorescent protein
LPS: Lipopolysaccharide
