# Supplementary material for: An analysis of intestinal morphology and incretin-producing cells using tissue optical clearing and 3-D imaging
Source: Sci Rep. 2022 Oct 20;12:17530. doi: 10.1038/s41598-022-22511-7 (PMC9584944; doi:10.1038/s41598-022-22511-7)
Supplement: Supplementary file 1 — Supplementary Figure 1. [file 41598_2022_22511_MOESM1_ESM.pdf]

Supplemental fig. 1

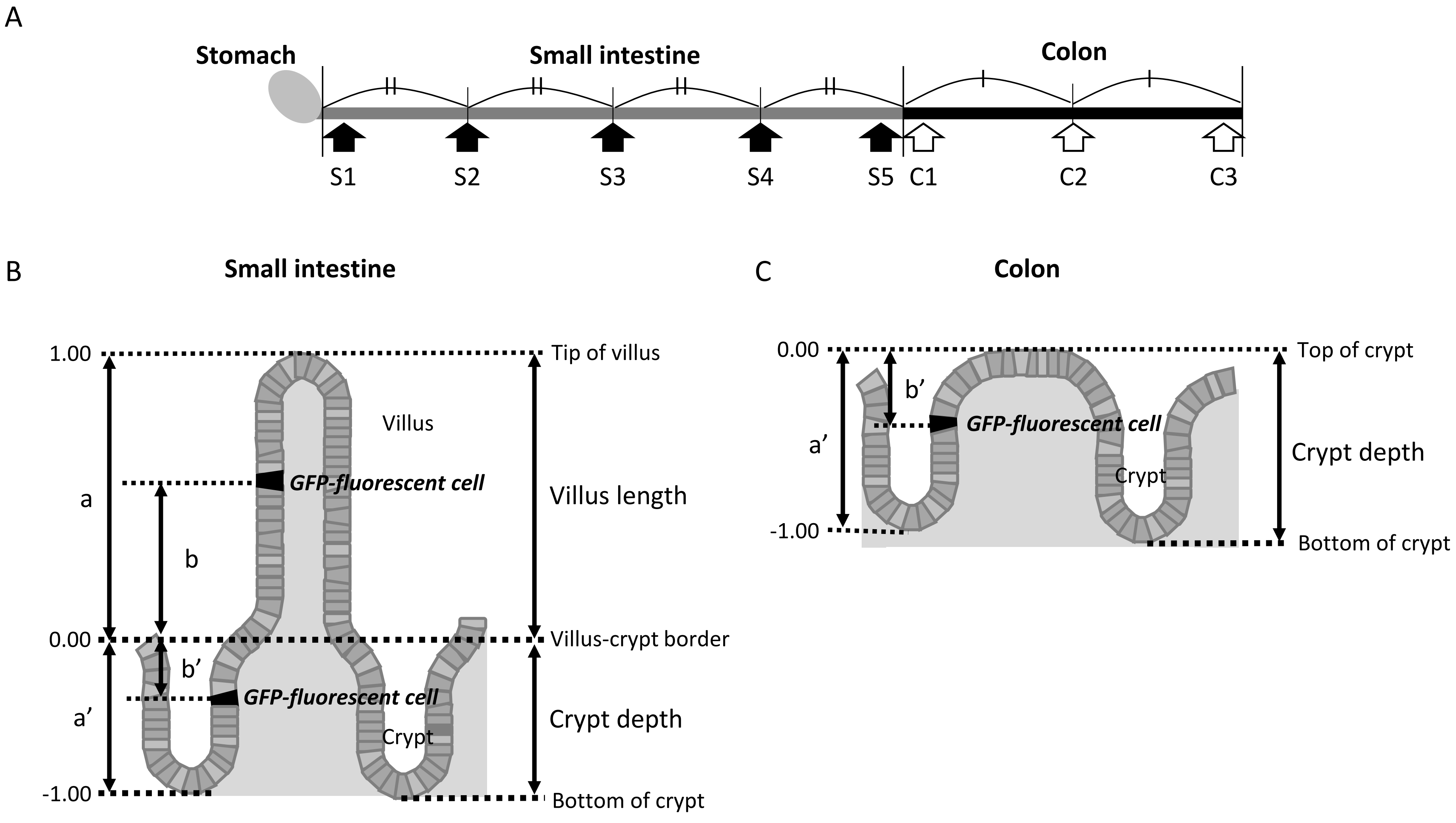

Supplemental figure 1. Intestinal samples and measuring method.

(A) Small intestine was divided into five parts (S1 to S5), while colon was divided into three parts (C1 to C3). Five small intestine and three colon samples were analyzed in this study. (B) Measurement of villus length and crypt depth in small intestine. The value of localization of incretin-producing cell in a villus was shown by b/a (Value of 1.00 and 0.00 indicates top of villus and villus-crypt border, respectively). The value of localization of incretin-producing cell in a crypt was shown by -b’/a’ (Value of 0.00 and -1.00 indicates villus-crypt border and bottom of crypt, respectively). (c) Measurement of crypt depth in colon. The value of localization of incretin-producing cell in a crypt was shown by -b’/a’ (Value of 0.00 and -1.00 indicates top of crypt and bottom of crypt, respectively).
